# Malfunctioning Plastic Biliary Endoprosthesis: Percutaneous Transhepatic Balloon Pulling Technique

**DOI:** 10.1155/2013/596480

**Published:** 2013-07-30

**Authors:** Umberto G. Rossi, Paolo Rigamonti, Maurizio Cariati

**Affiliations:** Department of Diagnostic Sciences, Division of Radiology and Interventional Radiology, San Carlo Borromeo Hospital, Via Pio II, 3, 20153 Milano, Italy

## Abstract

Percutaneous transhepatic removal techniques for malfunctioning plastic biliary endoprosthesis are considered safe and efficient second-line strategies, when endoscopic procedures are not feasible. We describe the percutaneous transhepatic balloon pulling technique in a patient with an unresectable malignant hilar cholangiocarcinoma.

## 1. Introduction

Plastic biliary endoprosthesis is successfully implanted as a minimally invasive palliative treatment in the management of malignant biliary obstruction [1–3]. Occlusion and/or migration of the plastic biliary endoprosthesis for tumour growth is a challenging procedure for gastroenterologist and interventional radiologist in removing the occluded and/or displaced endoprosthesis [1]. We present a case of occluded and proximal migrated plastic biliary endoprosthesis that was percutaneously removed by balloon catheter pulling technique.

## 2. Case Description

**Figure 1:**
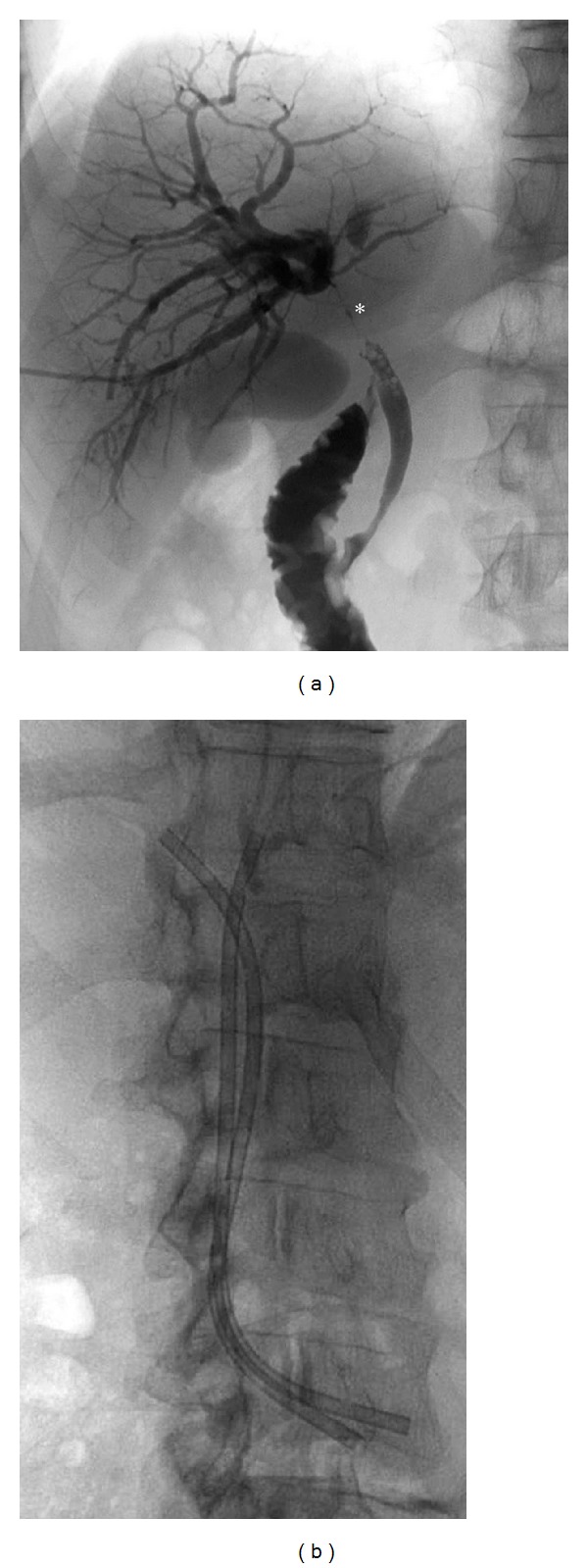
(a) Percutaneous cholangiography that demonstrates the hilum occlusion (∗) of the intrahepatic biliary ducts for the hilar cholangiocarcinoma (Klatskin tumor). (b) Bilateral plastic biliary endoprosthesis for the drainage of the right and left intrahepatic biliary ducts.

**Figure 2:**
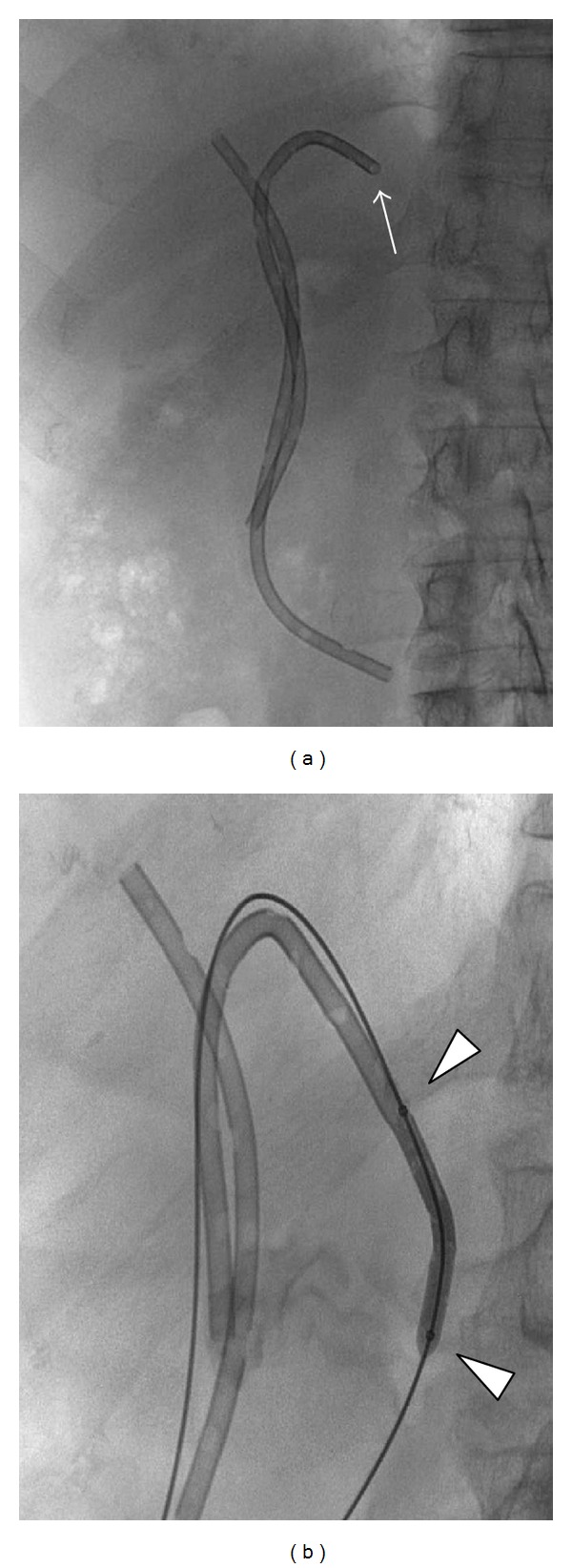
(a) Bilateral plastic biliary endoprosthesis: note the proximal migration of the left one (arrow). (b) The initial removal, by pulling back, of the plastic biliary endoprosthesis with the balloon catheter half inside the proximal part of the endoprosthesis: note the two radiopaque markers of the balloon catheter (arrowheads).

**Figure 3:**
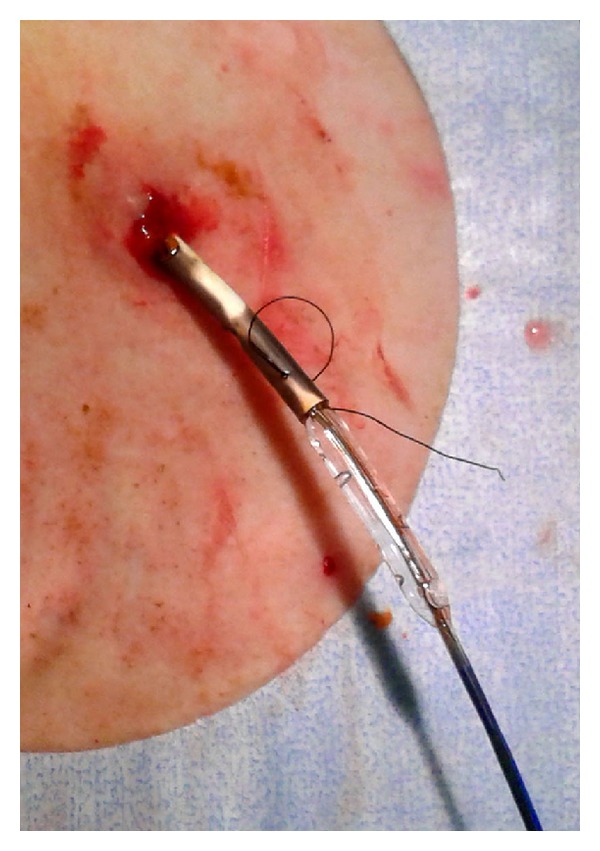
The almost complete removal of the plastic biliary endoprosthesis through the percutaneous access.

**Figure 4:**
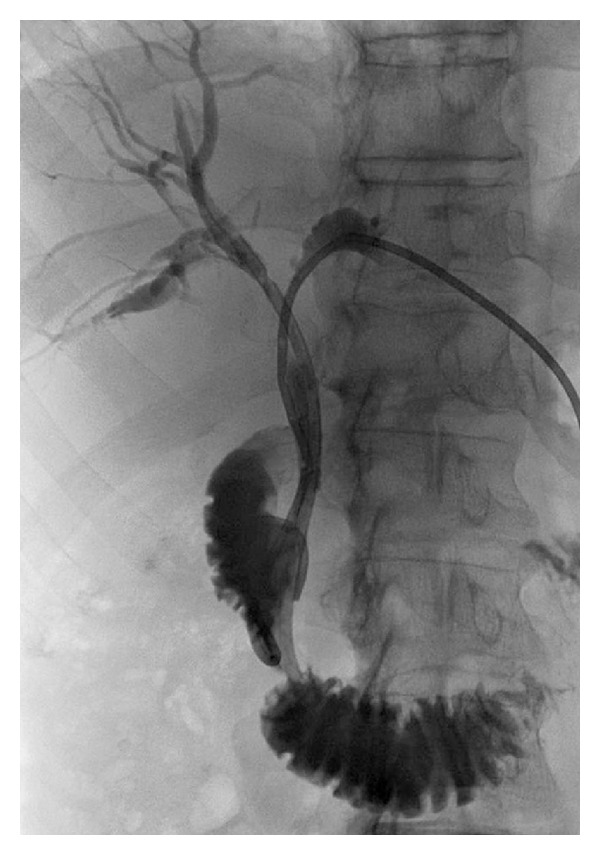
The end of the procedure with a percutaneous internal-external left biliary drainage and the native right plastic biliary endoprosthesis.

A 71-year-old female patient with an unresectable malignant hilar cholangiocarcinoma (Klatskin tumor) associated with a direct bilirubin level of 22 mg/dL and dilation of intrahepatic biliary ducts (Figure 1(a)) underwent palliative percutaneous bilateral external biliary drainage. After 5 days, the two external biliary drainages were replaced by two plastic biliary endoprostheses 12-Fr (Boston Scientific, Natick, MA), as a palliative treatment (Figure 1(b)). Three months later, she was evaluated for a mild dilatation of left intrahepatic biliary ducts and left percutaneous access biliary leak. The left biliary endoprosthesis appeared occluded at its proximal third and proximally migrated due to tumour growth (Figure 2(a)). Due to the proximal displacement of the left biliary endoprosthesis and the presence of the biliary leak, we decided to accede it percutaneously. After removing the left subcutaneous fixing ring, from the left percutaneous previous access (through the percutaneous biliary leak), we have passed a guide wire through the endoprosthesis from the proximal tip to first lateral hole because the endoprosthesis was occluded further this hole. An 8 Fr diameter introducer sheath (Terumo, Tokyo, Japan) was positioned. After multiple attempts of endoprosthesis removal by pushing it with the dilatator of the introducer sheath and a 3.5 × 40 mm balloon catheter (Abbott, Beringen, Switzerland) that was inflated coaxially outside the proximal end of the endoprosthesis, we decide to inflate the balloon catheter partially (half of it) inside the proximal end of the endoprosthesis (Figure 2(b)) and to pull back the device through the percutaneous transhepatic access (Figure 3). No friction and complications occurred during the manoeuvre. Patient underwent percutaneous internal-external left biliary drainage (Figure 4) and consequently a new plastic biliary endoprosthesis.

## 3. Discussion

Malfunctioning plastic biliary endoprosthesis is managed by replacing the occluded, kinked, or migrated endoprosthesis with a new one. There are numerous endoscopic and interventional radiologic techniques to remove occluded and/or migrated plastic biliary endoprostheses [1, 2]. For each case, the best technique, route, and device have to be chosen. When endoscopic procedures are not feasible, the percutaneous transhepatic interventional radiologic techniques are the first-line approach in removing the occluded plastic biliary endoprosthesis [1, 3]. Several different interventional radiologic techniques have been described in literature for removing the malfunctioning plastic biliary endoprosthesis. These techniques include dislodge of the plastic biliary endoprosthesis by pushing it into the bowel with: (i) a long introducer or a new drainage, (ii) a balloon catheter that is inflated coaxially with the endoprosthesis, (iii) a balloon catheter that is inflated parallel to the endoprosthesis, and (iv) a goose-neck snare at the proximal or distal end; and by pulling it out from the transhepatic tract with a large-diameter introducer sheath and a goose-neck snare [1–5]. No clear data exist about the superiority of one technique over others.

In our cases, the endoscopic procedures were not feasible due to the proximal migration of the endoprosthesis, and also the four pushing techniques were not suitable for the complete occlusion of the endoprosthesis and its external compression due to tumour growths. The pulling technique was considered our first choice. So, before using the traditional pushing approach with a goose-neck snare, we have tried to do the same manoeuvre with the already-positioned balloon catheter. Then, from the 8 Fr introducer sheath, the balloon catheter with almost the same diameter of the endoprosthesis was inflated half inside the proximal end of the endoprosthesis and the other half outside. This gave us two technical advantages: first a firm grip between the endoprosthesis and the balloon catheter and second a tapered new tip at the proximal end of the endoprosthesis. 

To our knowledge, the use of a balloon catheter as a device for pulling out an occluded plastic biliary endoprosthesis through the percutaneous transhepatic access has not been illustrated previously in literature.

In conclusion, the knowledge of all different percutaneous interventional radiologic techniques with their devices helps the interventional radiologist to achieve the procedural and the clinical success.
